# Prognostic Value of Resting Energy Expenditure Measured by Indirect Calorimetry in Patients with Cirrhosis Referred for Liver Transplantation †

**DOI:** 10.3390/nu17233709

**Published:** 2025-11-26

**Authors:** Natalie L. Y. Ngu, Georgia Knapp, Helen Vidot, Joanne Craik, Rachael Jacob, Simone I. Strasser, Geoffrey W. McCaughan, Ken Liu

**Affiliations:** 1 AW Morrow Gastroenterology and Liver Centre, Royal Prince Alfred Hospital, Camperdown, NSW 2050, Australia; natalie.ngu@monash.edu (N.L.Y.N.); helen.vidot@health.nsw.gov.au (H.V.); rachael.jacob@health.nsw.gov.au (R.J.);; 2 Faculty of Medicine, Nursing, and Health Sciences, Monash University, Clayton, VIC 3800, Australia; 3 Department of Nutrition & Dietetics, Royal Prince Alfred Hospital, Camperdown, NSW 2050, Australia; 4 Faculty of Medicine and Health, University of Sydney, Camperdown, NSW 2050, Australia

**Keywords:** malnutrition, liver transplantation, cirrhosis, indirect calorimetry

## Abstract

Background: Malnutrition is common in cirrhosis but is challenging to identify, grade, and manage during liver transplantation (LT) assessment. The resting energy expenditure informs nutritional needs and can be measured with indirect calorimetry (IC); however, it is not routinely used in this context. We aimed to assess the prognostic value of the measured resting energy expenditure (mREE) and its associations in patients with cirrhosis referred for LT assessment. Methods: We performed a single-center, retrospective cohort study of adult patients with cirrhosis who underwent IC between 2002 and 2019 at a statewide LT center. The predicted REE (pREE) was estimated using the Harris–Benedict equation. Patients were classified as normo-, hypo-, and hypermetabolic based on the difference between the mREE and pREE. Malnutrition was determined prospectively using the Subjective Global Assessment. The primary outcome was LT-free survival. Results: A total 203 patients were recruited (74% male, median age 55 [IQR 49–60], median MELD score 14 [IQR 11–17]). The most common cause of cirrhosis was alcohol (40%). The median pREE and mREE were 1652 (IQR 1459–1873) and 1708 (IQR 1490–1907) kcal/day, respectively. The mREE was lower in females and those with older age and malnutrition, significantly correlating with body composition measures (*p* < 0.01 for all). Most patients were normometabolic (88.6%), with others being hypometabolic (3.5%) or hypermetabolic (8.0%). After a median follow-up of 104 months (IQR 28–175), there were 107 LT and 49 deaths without LT. Hypermetabolism was independently associated with a worse LT-free survival (HR 2.11, 95%CI 1.161–3.845, *p* = 0.014) but, along with mREE, had no impact on post-LT graft survival. Conclusions: Patients with cirrhosis and hypermetabolism identified with IC had a two-fold increased risk of LT or death, independent of the MELD score and nutritional status. These findings suggest that IC provides valuable prognostic information for pre-LT assessment and may support individualized nutritional and risk stratification strategies in cirrhosis.

## 1. Introduction

Malnutrition is common among patients with cirrhosis referred for liver transplantation (LT) [1,2]. The underlying cause of malnutrition in this cohort is multifactorial, including the catabolic effects of cirrhosis, anorexia, malabsorption, and sociodemographic and physiological contributors to malnutrition [3]. Optimizing nutritional status before LT is critical due to its impact on surgical risk and post-operative recovery. Malnutrition and sarcopenia are well-known predictors of adverse post-transplant outcomes including infection, prolonged mechanical ventilation, and longer intensive care unit and hospital length of stay [2,4]. Malnutrition is a modifiable risk factor that can be addressed pre-operatively, offering a valuable opportunity to improve outcomes.

The accurate evaluation of malnutrition in this cohort remains challenging [5]. Conventional nutritional assessments—including body weight, serum albumin, and handgrip strength—are often confounded by fluid retention, hepatic synthetic dysfunction, and variability in equipment or measurement protocols [6,7]. As a result, malnutrition may be under-recognized or inadequately addressed prior to LT, potentially compromising outcomes. While malnutrition is prevalent pre-transplant, weight gain and features of metabolic syndrome are also common post-transplant, further complicating the nutritional management of these patients [8,9].

Indirect calorimetry (IC) is a non-invasive tool that measures the resting energy expenditure (REE) based on pulmonary gas exchange, offering an objective measure of an individual’s nutritional requirements [10]. IC has been validated in various clinical settings and is more accurate than predictive equations in determining energy requirements in patients with cirrhosis [11]. Additionally, traditional anthropometric and biochemical assessments of nutrition are confounded by ascites [12] and hepatic dysfunction [13]. Despite this, IC is not routinely used in transplant assessment due to time restraints, resources, or limited availability [14].

**Figure 1:**
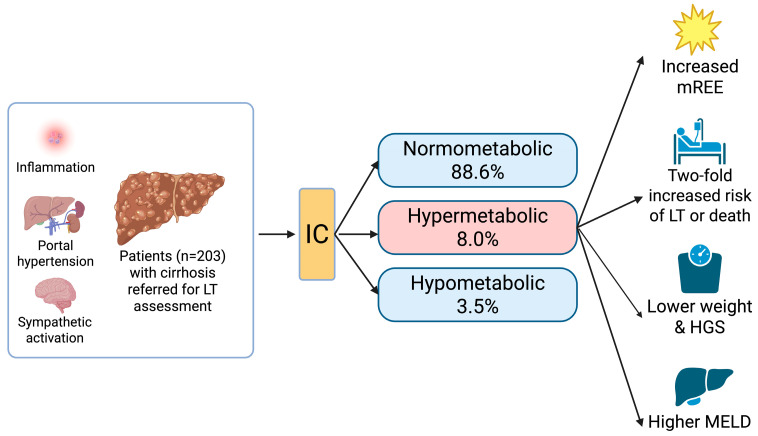
Patients referred for LT assessment with hypermetabolism identified with IC had a 2.11 increased risk of LT or death compared to hypo- or normometabolic patients [19]. HGS = handgrip strength, IC = indirect calorimetry, LT = liver transplantation, MELD = Model for End-Stage Liver Disease, mREE = measured resting energy expenditure. Created in BioRender. Ngu, N. (2025) [19]. https://BioRender.com/drh96tp, accessed on 26 October 2025.

Given the importance of nutritional status in influencing perioperative risk and long-term outcomes following LT, there is a need for more reliable and standardized assessment tools [15]. The existing literature demonstrates the feasibility of IC in this setting, but data evaluating the associations between pre-transplant REE and clinically significant transplant outcomes such as survival remain limited and largely constrained to small cohorts [16,17,18]. Therefore, this study aimed to evaluate the prognostic significance of REE measured by IC in patients with cirrhosis referred for LT assessment at a single Australian center (Figure 1). The results of this study may support the broader implementation of IC as a standardized tool for routine nutritional assessment in LT evaluation.

## 2. Materials and Methods

We performed a single-center, retrospective cohort study of all adults with cirrhosis referred to a tertiary referral LT center who underwent IC as part of pre-LT assessment between 2002 and 2019. Patients were eligible if they underwent at least one measurement of REE via IC assessment as part of routine clinical assessment. Patients were excluded if IC was not performed or if they did not attend the LT assessment clinic.

Data were obtained through a retrospective review of a prospectively maintained transplant database, supplemented by additional information extracted from electronic medical records and archived paper-based files.

The severity of liver disease was evaluated using the Child–Pugh and Model for End-Stage Liver Disease (MELD) scores. All patients were evaluated by experienced clinical dietitians as part of standard pre-transplant workup. Anthropometric assessments were performed, including handgrip strength (HGS), mid-arm circumference (MAC), mid-arm muscle circumference (MAMC), tricep skin fold thickness (TSFT), height, and weight. A Subjective Global Assessment (SGA) was also performed prospectively to classify patients as well-nourished (SGA-A), moderately malnourished (SGA-B), or severely malnourished (SGA-C). Patients were advised on dietary optimization and offered nutritional supplementation as appropriate. All patients were offered IC as part of routine nutritional assessment, although a small number declined testing due to factors such as claustrophobia, time constraints, or anticipated breathing difficulties.

IC was performed using the Delta Trac II (Datex-Ohmeda) system from 2003 to 2012, when it was discontinued, and the COSMED Quark system from 2012 to 2019. Equivalence has been demonstrated between the two machines [20], and a recent study reported that the Q-NRG indirect calorimeter used in canopy mode performs similarly to the Delta Trac II device [21]. Both devices were calibrated regularly in line with the manufacturer’s recommendations. Assessments took place in the morning after a 10–12 h fasting period. Patients were studied in a quiet, private room with subdued lighting to minimize stimulation. The first 10 min of recorded data were excluded to allow patients to rest. Patients were instructed to remain awake throughout the test. The test continued until a steady state measurement was obtained, defined as a five-minute period during which the coefficient variation for both oxygen consumption (VO_2_) and carbon dioxide production (VCO_2_) was less than 10%. Fuel utilization was calculated using an average urinary nitrogen excretion of 12.3 g/day, based on prior 24 h urine studies in 74 patients with cirrhosis from our unit, analyzed using the Kjeldahl methodology.

Predicted REE (pREE) was calculated using the Harris–Benedict equations [22,23]. For males, the equation used was pREE (kcal/day) = 66 + (13.75 × weight in kg) + (5.0 × height in cm) − (6.76 × age in years). For females, the equation was pREE (kcal/day) = 655 + (9.56 × weight in kg) + (1.85 × height in cm) − (4.68 × age in years). Patients were classified as normo-, hypo-, and hypermetabolic based on the difference between measured REE (mREE) and pREE, with thresholds of ±20%, <20%, and +20%, respectively [11,24,25,26]. 

This article is a revised and expanded version of a conference abstract entitled “Prognostic value of measuring resting energy expenditure by indirect calorimetry in cirrhosis patients referred for liver transplantation”, which was presented at the World Congress of Gastroenterology, Melbourne, Australia, September 2025 [27].

### Statistical Analysis

Continuous variables were expressed in means with standard deviation (SD) or medians with interquartile range (IQR) as appropriate. Differences between subgroups were analyzed using χ^2^ or Fisher exact tests for categorical variables and Student’s *t*-test or the Mann–Whitney U test for continuous variables as appropriate. Correlations between sarcopenia and frailty and different variables were determined through Spearman’s correlation coefficient analysis. The primary outcome of interest was transplant-free survival (i.e., time to death or LT), and secondary outcomes were overall survival on the waitlist, graft survival post-LT, and overall survival post-LT. Survival was estimated using the Kaplan–Meier method, with a log-rank test to determine statistical significance between study groups. Univariable analyses were conducted on collected variables to determine clinical parameters associated with outcomes. Multicollinearity between covariates was assessed with a variance inflation factor (VIF); values exceeding five were deemed significant. A multivariable Cox regression model using a backward stepwise approach was performed among covariates that were associated with death or LT in univariable analysis (*p* < 0.1). The hazard ratio (HR) and 95% confidence interval (CI) of the risk factors were calculated. All statistical analyses were conducted using Stata version 16 (StataCorp, College Station, TX, USA).

The study was conducted according to the Declaration of Helsinki and was approved by the Sydney Local Health District Human Ethics Research Committee (X23-0094 and 2023/ETH00431).

## 3. Results

### 3.1. Patient Characteristics

A total of 203 patients were included in the study. The majority were male (73.9%), the median age was 55 years (IQR: 49–60), and the most frequent cirrhosis etiology was alcohol (40.4%). The median baseline MELD was 14 (IQR: 11–17), and 19.2% had concomitant hepatocellular carcinoma (HCC). At the time of baseline nutritional assessment, 44.3% had ascites. The nutritional assessment results demonstrate that malnutrition was present in most patients, classified as SGA-C with severe malnutrition in 18.6%, SGA-B with moderate malnutrition in 53.1%, and SGA-A considered well-nourished in 28.4%. The baseline characteristics are shown in Table 1.

### 3.2. Calorimetry

The median mREE was 1708 (IQR: 1490–1907) kcal/day and the median pREE was 1652 (IQR: 1459–1873) kcal/day. Height, weight, BMI, MAC, MAMC, TSFT, and grip strength all directly and significantly correlated with mREE, while increasing age was inversely correlated (Table 2). Males had a significantly higher mREE compared to females (1748 [IQR: 1597–1946] vs. 1457 [IQR: 1246–1687] kcal/day, *p* < 0.001). The measured REE was significantly lower in those with worse malnutrition compared to normal nutrition (1877 [IQR: 1659–2086] kcal/day in SGA-A vs. 1691 [IQR: 1456–1880] kcal/day in SGA-B *p* < 0.001 and vs. 1520 [IQR: 1383–1717] kcal/day in SGA-C, *p* < 0.001; both comparisons remain significant with Bonferroni correction).

Based on the mREE and pREE, patients were categorized as normometabolic (88.6%), hypometabolic (3.5%), or hypermetabolic (8.0%), with the distribution of characteristics shown in Table 3. According to the definition, hypermetabolic patients had a numerically lower pREE and higher mREE. In general, they appeared more likely be female and lighter, with lower anthropometry measurements and higher MELD scores compared to normo- or hypometabolic patients. However, not all of these comparisons reached statistical significance due to the Bonferroni correction for multiple comparisons and low numbers of hypometabolic patients (Table 3).

### 3.3. Patient Outcomes

Over a median follow-up of 104 months (IQR: 28–175), a total of 117/203 (57.6%) patients progressed to LT waitlisting, with the remaining 86 excluded due to improvements in condition, ineligibility, or death. Waitlist mortality was 10/117 (8.5%). Liver transplantation was performed in 107/203 (52.7%) patients, and there were 49/203 (24.1%) total deaths without LT.

**Figure 2:**
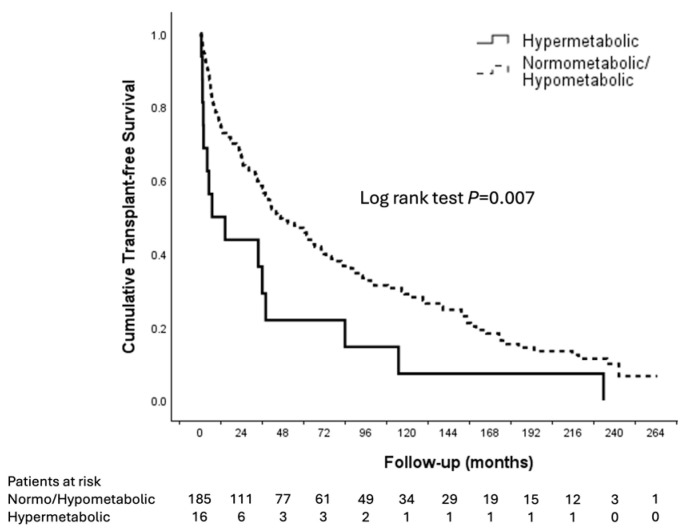
Survival analyses. Kaplan–Meier analyses of liver transplant-free survival in hypermetabolic vs. normometabolic or hypometabolic patients.

Patients with hypermetabolism exhibited significantly worse transplant-free survival compared to normo- or hypometabolism in the Kaplan–Meier analysis (Figure 2). After adjusting for other variables through Cox regression analysis, hypermetabolism remained an independent predictor of LT or death (aHR 2.11, 95% CI 1.161–3.845, *p* = 0.014) (Table 4). A higher mREE was also significantly associated with LT or death (aHR 1.081 per 100 kcal/day increase, 95% CI 1.017–1.148, *p* = 0.013). Other independent predictors included the presence of concomitant HCC (aHR 1.69, 95% CI 1.125–2.523, *p* = 0.011), a higher MELD score (aHR 1.068 per point increase, 95% CI 1.040–1.098, *p* < 0.001), and a lower MAMC (aHR 0.995, 95% CI 0.991–0.998, *p* = 0.003). A higher pREE also predicted LT or death (aHR 1.001 per kcal/day increase, 95% CI 1.000–1.001, *p* = 0.006) but had a significant collinearity with mREE (Supplementary Table S1). Neither metabolism status (hypo-, normo-, or hypermetabolism) nor mREE were predictors of waitlist mortality (*p* > 0.4 for all).

Among the 107 patients who received LT, 5/107 (4.7%) required re-transplantation and 41/107 (38.3%) died post-LT. Neither metabolism status nor mREE predicted for graft or patient survival (*p* > 0.4 for all). Metabolic status and mREE were also not significantly associated with intensive care unit and total hospital length of stay post-LT (*p* > 0.2 for all).

## 4. Discussion

Malnutrition is a common complication of cirrhosis [2], but remains challenging to objectively diagnose and treat. In this retrospective study of patients who underwent IC assessment following a referral for LT work-up, we found that a higher mREE and hypermetabolism were associated with a poorer transplant-free survival, independent of MELD and nutritional status, highlighting the prognostic value of IC in this cohort beyond conventional liver- and nutrition-specific assessment.

The reported prevalence of hypermetabolism in cirrhosis ranges from 8 to 58% [28,29,30], with the wide range attributed to diagnostic criteria, including 115–120% pREE and the correction for fluid overload/fat-free mass, population heterogeneity, liver disease severity, and nutritional status [11]. Hypermetabolism in cirrhosis is associated with portal hypertension, ascites, increased sympathetic nervous system activation, higher fat-free mass, and insulin resistance [26,29,31], and with liver disease severity [24,26]. These factors are associated with raised pro-inflammatory and anti-inflammatory cytokines [32] and enterocytic mitochondrial dysfunction [33], driving hypermetabolism in cirrhosis. Compared to general cirrhosis populations, hypermetabolism was less prevalent in our cohort [24], which may reflect selection bias in those referred for LT assessment compared to critically ill cirrhosis populations in the literature. By the nature of potential transplant candidacy, these patients have a smaller proportion with alcohol-related liver disease, and few still actively drink; this group is less likely to have significant co-morbidities or include those with advanced age. Indeed, our 8% prevalence of hypermetabolism is consistent with other studies of patients referred for LT assessment with similar baseline MELD scores using the same threshold of >120% pREE [24]. We found that an increasing malnutrition, determined through SGA category, was inversely associated with the median mREE, consistent with previously published evidence in a LT waitlist cohort [28], and attributed to mechanisms such as the reduction in fat-free mass, including muscle, prioritizing energy conservation [34]. Accordingly, an increased REE in cirrhosis patients has been identified when adjusted for fat-free mass, measured by dual-energy X-ray absorptiometry [29]. Hence, the accurate identification of hypermetabolism in this cohort may influence individualized dietary recommendations to address higher energy expenditure and help prioritize patients on the waiting list (in addition to MELD score), due to its independent prognostic associations with transplant-free survival. The integration of IC into routine pre-LT assessment could allow the prioritization of high-risk, hypermetabolic patients for targeted nutritional interventions. However, barriers to implementation include resource limitations, due to equipment, consumables, and staffing availability, and costs. Encephalopathy can interfere with readings, as can variability with acute phase conditions such as sepsis. The accuracy of readings in patients who are intubated or receiving non-invasive ventilation in acute liver failure is compromised. Therefore, further robust evidence is required to justify the routine implementation of IC, along with careful patient selection.

Interestingly, hypermetabolism and mREE were not predictive of post-LT outcomes in our study. This may reflect the resolution of cirrhosis-related drivers of hypermetabolism following transplantation, including portal hypertension, systemic inflammation, and muscle catabolism, which may contribute to the normalization or near-normalization of REE [35,36]. Nutritional recovery and weight gain after LT, together with the dominant influence of perioperative complications, graft function, infection, and immunosuppression on post-LT outcomes, may diminish the prognostic impact of pre-LT energy expenditure. Furthermore, the relatively small proportion of hypermetabolic patients in our cohort may have limited power to detect post-LT associations. Similarly, with a waitlist mortality of only 8.5% (10 patients), we were unable to show that hypermetabolism was significantly associated with death on the waitlist. Instead, its influence on transplant-free survival in our cohort was predominately focused on predicting those who required LT. Nonetheless, our findings suggest that, while hypermetabolism is an important prognostic marker in the pre-transplant setting, its relevance diminishes once the underlying liver disease is removed.

The diagnosis and quantification of malnutrition in cirrhosis remains a clinical challenge despite the availability of clinical tools including the SGA and Royal Free Hospital–Nutritional Prioritizing Tool, as these require specific clinician training, the availability of resources and time, were developed for screening rather than diagnosis, and are subject to inter-observer variability [37,38]. IC addresses these limitations through the provision of objective and comparable readings. Given the well-established associations between malnutrition and both mortality [39] and post-transplant outcomes [2], an accurate determination of energy requirements is crucial for the provision of appropriate treatment plans. The historical calculation of energy requirements has relied on predictive equations using clinical data including age, sex, weight, and height [22,40], and remains useful due to its simplicity, accessibility, and low cost. However, a significant limitation is the poor agreement between energy requirements calculated by predictive equations and energy expenditure measured using indirect calorimetry, particularly in intensive care unit [41] and cirrhosis patients [6,42]. As demonstrated in our study, pREE largely underestimates energy requirements in cirrhosis [6,43], attributed to factors including the presence of ascites [42], which has been found to increase resting metabolic rate [12], as well as cirrhosis-specific alterations in body composition and systemic inflammatory processes [30]. The measurement of REE using IC can therefore overcome some of these challenges; however, limitations include time requirements, expense, clinician training, and the accessibility of equipment [14,44]. Additionally, the accuracy and practical utilization of IC assessment are limited in critical illness, supplemental oxygen, nausea, mask intolerance, and cognitive dysfunction, such as encephalopathy [10]. However, our study results suggest that, in this cohort of patients referred for LT assessment, IC may be prognostically helpful in identifying those with hypermetabolism at an increased risk of LT or death. Therefore, targeting this subset of patients with evidence-based strategies for both nutritional (e.g., more aggressive caloric prescription) and more frequent clinical monitoring is warranted.

Our study has several limitations, including its single site, modest patient numbers, and retrospective nature. There is the potential for selection bias due to the incomplete recruitment of IC amongst all patients undergoing transplantation work-up. This was addressed by combining research databases to increase sample size, but we recognize that we are unable to closely examine the characteristics of those “opting out” of IC in this retrospective study. Body mass index and weight were taken from the medical records, and the fluid status at the time of measurement was not recorded, which is of significance both due to weight fluctuations and also the association between ascites and an increased REE [12]. We did not collect data on changes in dietitian recommendations following IC assessments, which may have influenced the subsequent nutritional status and outcomes. Future multicenter prospective studies should validate the prognostic value of IC and assess whether IC-guided nutritional interventions improve survival and LT outcomes.

## 5. Conclusions

REE, measured using IC, independently predicted LT-free survival, identifying hypermetabolic patients at an increased risk of LT or death. These findings support integrating IC into routine pre-LT assessment for risk stratification and personalized nutritional care. Incorporating IC into pre-LT evaluation may refine patient prioritization and support the design of targeted, nutrition-based interventions in cirrhosis.

## Figures and Tables

**Table 1:** Baseline characteristics of patient cohort.

Characteristic	Result *n* (%) or Median (IQR)
Male sex	150 (73.9)
Median age (years)	55 (49–60)
Median height (m)	1.72 (1.67–1.78)
Median weight (kg)	79 (69–93)
Median BMI (kg/m^2^)	27.0 (23.7–31.1)
Cirrhosis cause	
- Alcohol	82 (40.4)
- MASLD	62 (30.5)
- HCV	33 (16.3)
- HBV	18 (8.9)
- Other	8 (3.9)
Concomitant HCC	39 (19.2)
Median MELD score	14 (11–17)
Ascites	90 (44.3)
Encephalopathy	73 (36.0)
Subjective Global Assessment	
- A (well nourished)	55 (28.4)
- B (moderate malnutrition)	103 (53.1)
- C (severe malnutrition)	36 (18.6)
Median mid-arm circumference (cm)	29.5 (26.5–34.6)
Median mid-arm muscle circumference (cm)	25.5 (22.7–28.1)
Grip strength (kg/mmHg)	
- Left	27.0 (20.7–33.3)
- Right	29.2 (22.5–36.7)
Tricep skin fold thickness (mm)	12.8 (8.6–22.1)
Predicted resting energy expenditure (kcal/day)	1652 (1459–1873)
Measured resting energy expenditure (kcal/day)	1708 (1490–1907)
Metabolism	
- Normometabolic	178 (88.6)
- Hypometabolic	7 (3.5)
- Hypermetabolic	16 (8.0)

Data presented as n (%) or median (IQR). BMI = body mass index; HBV = hepatitis B virus; HCC = hepatocellular carcinoma, HCV = hepatitis C virus, IQR = interquartile range; kg = kilograms, kcal = kilocalories; m^2^ = meters squared; MASLD = metabolic dysfunction-associated steatotic liver disease; mm = millimeters.

**Table 2:** Significant correlations with measured resting energy expenditure.

Variable	Spearman Rho	*p*
Age	−0.209	0.003
Height	0.574	<0.001
Weight	0.645	<0.001
BMI	0.431	<0.001
MAC	0.458	<0.001
MAMC	0.500	0.002
Grip strength L	0.498	<0.001
Grip strength R	0.509	<0.001
TSFT	0.172	0.015

BMI = body mass index; MAC = mid-arm circumference; MAMC = mid-arm muscle circumference; TSFT = tricep skin fold thickness.

**Table 3:** Baseline characteristics according to metabolism category.

Characteristic	Normometabolic *n* = 178	Hypometabolic *n* = 7	Hypermetabolic *n* = 16	*p* *
Male sex (%)	138 (77.5)	6 (85.7)	6 (37.5)	0.002 ^a^
Age (year)	55 (48–60)	55 (53–57)	56 (52–66)	0.311
Height (m)	1.74 (1.67–1.79)	1.73 (1.65–1.75)	1.67 (1.60–1.75)	0.078
Weight (kg)	80.0 (69.4–92.5)	101.7 (70.0–107.4)	74.5 (60.0–78.5)	0.036
BMI (kg/m^2^)	26.9 (23.8–31.0)	32.5 (25.7–36.3)	26.3 (23.0–28.9)	0.112
MELD	14 (11–17)	13 (12–15)	20 (13–24)	0.051 ^b^
Concomitant HCC (%)	37 (20.6)	0 (0)	2 (12.5)	0.311
Ascites (%)	97 (54.2)	3 (42.9)	10 (62.5)	0.896
Encephalopathy (%)	63 (35.2)	5 (71.4)	4 (28.6)	0.123
MAC (cm)	29.8 (26.6–35.0)	35.0 (27.9–38.5)	27.0 (25.0–29.3)	0.008 ^c^
MAMC (cm)	25.6 (22.7–28.2)	25.2 (23.6–30.2)	23.5 (21.4–25.6)	0.059
TSFT (mm)	12.8 (8.7–22.9)	21.5 (13.6–29.3)	9.9 (7.7–15.0)	0.033
Grip strength L	28.0 (22.0–38.5)	25.7 (19.3–32.0)	19.7 (16.9–24.9)	0.010 ^d^
Grip strength R	30.3 (23.3–36.7)	26.3 (13.6–29.3)	24.9 (17.1–27.0)	0.021 ^e^
SGA A B C	52 (29.9) 91 (52.3) 31 (17.8)	2 (28.6) 5 (71.4) 0 (0)	2 (13.3) 8 (53.3) 5 (33.3)	0.294
Predicted REE (kcal/day)	1662 (1471–1888)	1967 (1503–2100)	1418 (1342–1652)	0.004 ^f^
Measured REE (kcal/day)	1708 (1500–1903)	1404 (1183–1540)	1822 (1658–2062)	0.001 ^g^
Difference between predicted and measured REE (kcal/day)	21 (−128–146)	−496 (−562–−320)	352 (320–457)	<0.001 ^h^

* With Bonferroni correction for multiple comparisons, *p* < 0.017 is considered significant here: ^a^ Comparison for sex is significant for normometabolic vs. hypermetabolic (*p* = 0.016). ^b^ Comparison for MELD is significant for normometabolic vs. hypermetabolic (*p* = 0.016). ^c^ Comparison for MAC is significant for normometabolic vs. hypermetabolic (*p* = 0.007) and hypometabolic vs. hypermetabolic (*p* = 0.007). ^d^ Comparison for grip strength L is significant for normometabolic vs. hypermetabolic (*p* = 0.003). ^e^ Comparison for grip strength R is significant for normometabolic vs. hypermetabolic (*p* = 0.007) ^f^ Comparison for predicted REE is significant for normometabolic vs. hypermetabolic (*p* = 0.002). ^g^ Comparison for measured REE is significant for hypometabolic vs. hypermetabolic (*p* < 0.001). ^h^ Comparison for difference in predicted and measured REE is significant for normometabolic vs. hypermetabolic (*p* < 0.001) and hypometabolic vs. hypermetabolic (*p* < 0.001). Data presented as n (%) or median (IQR): BMI = body mass index; HCC = hepatocellular carcinoma; kg = kilograms; m = meters; m^2^ = meters squared; MAC = mid-arm circumference; MAMC = mid-arm muscle circumference; MELD = Model for End-Stage Liver Disease score; REE = resting energy expenditure; SGA = Subjective Global Assessment; TSFT = tricep skin fold thickness.

**Table 4:** Univariable and multivariable predictors in Cox regression analysis of death or liver transplant.

	Univariable			Multivariable		
	HR	95% CI	*p*-Value	aHR	95% CI	*p*-Value
Male sex (vs. female)	1.349	0.932–1.952	0.112	1.072	0.614–1.873	0.806
HCC (yes vs. no)	1.341	0.92–1.938	0.118	1.685	1.125–2.523	0.011
MELD (per point increase)	1.065	1.039–1.091	<0.001	1.068	1.040–1.098	<0.001
MAC (per cm increase)	0.998	0.995–1.001	0.181	1.004	0.996–1.013	0.329
MAMC (per cm increase)	0.996	0.992–0.999	0.020	0.995	0.991–0.998	0.003
TSFT (per mm increase)	0.985	0.967–1.003	0.097	0.991	0.960–1.024	0.598
SGA						
A (Ref)	1			1		
B	1.403	0.962–2.045	0.079	1.356	0.866–2.125	0.183
C	1.663	1.010–2.738	0.045	1.394	0.765–2.539	0.278
REE measured (per 100 kcal/day increase)	1.053	0.999–1.109	0.055	1.081	1.017–1.148	0.013
Metabolism						
Normometabolic (Ref)	1			1		
Hypometabolic	1.258	0.553–2.860	0.584	2.204	0.917–5.302	0.078
Hypermetabolic	2.070	1.211–3.537	0.008	2.113	1.161–3.845	0.014

HCC = hepatocellular carcinoma; MAC = mid-arm circumference; MAMC = mid-arm muscle circumference; MELD = Model for End-Stage Liver Disease score; REE = resting energy expenditure; Ref = reference; SGA = Subjective Global Assessment; TSFT = tricep skin fold thickness.

## Data Availability

The data presented in this study are available on request from the corresponding author due to privacy and ethical restrictions.

## Supplementary Materials

The following supporting information can be downloaded at https://www.mdpi.com/article/10.3390/nu17233709/s1. Table S1: Variance inflation factors of multivariable analysis with (a) and without (b) predicted REE in the model. Table S2: Univariable and multivariable predictors in Cox regression model of death or liver transplant after removing both predicted REE and measured REE indicators as requested by reviewers.

## Abbreviations

The following abbreviations are used in this manuscript:

CI	Confidence interval
HCC	Hepatocellular carcinoma
HGS	Handgrip strength
HR	Hazard ratio
IC	Indirect calorimetry
IQR	Interquartile range
LT	Liver transplantation
MAC	Mid-arm circumference
MAMC	Mid-arm muscle circumference
MASLD	Metabolic dysfunction-associated steatotic liver disease
MELD	Model for End-Stage Liver Disease
mREE	Measured resting energy expenditure
pREE	Predicted resting energy expenditure
REE	Resting energy expenditure
SD	Standard deviation
SGA	Subjective Global Assessment
TSFT	Tricep skin fold thickness
VIF	Variance inflation factor
VCO_2_	Coefficient variation of carbon dioxide production
VO_2_	Coefficient variation of oxygen production
