# Supplementary material for: Prognostic Value of Resting Energy Expenditure Measured by Indirect Calorimetry in Patients with Cirrhosis Referred for Liver Transplantation
Source: Nutrients. 2025 Nov 26;17(23):3709. doi: 10.3390/nu17233709 (PMC12694111; doi:10.3390/nu17233709)
Supplement: Supplementary file 1 [file nutrients-17-03709-s001.zip › nutrients-3978782-supplementary.pdf]

Supplementary Table S1 – Variance inflation factors of multivariable analysis with (a) and without (b) Predicted REE in the model

(A)

Model	Collinearity Statistics Tolerance	Collinearity Statistics VIF
(Constant)		
sex	.442	2.262
HCC	.877	1.140
MELD	.862	1.161
MAC	.238	4.759
MAMC	.482	2.075
TSF	.249	4.022
SGA	.526	1.900
**Predicted REE**	**.186**	**5.374**
Measured REE	.370	2.706
Metabolism	.812	1.231

(B)

Model	Collinearity Statistics Tolerance	Collinearity Statistics VIF
(Constant)		
sex	.612	1.635
HCC	.922	1.085
MELD	.870	1.149
MAC	.243	4.140
MAMC	.483	2.070
TSF	.255	3.914
SGA	.528	1.893
Measured REE	.587	1.704
Metabolism	.832	1.202

Supplementary Table S2. Univariable and multivariable predictors on cox regression model of death or liver transplant after removing both Predicted REE and Measured REE indicators as requested by reviewers

	**Univariable**			**Multivariable**		
	HR	95% CI	P-value	aHR	95% CI	P-value
Male sex (vs. female)	1.349	0.932-1.952	0.112	1.422	0.954-2.119	0.084
HCC (yes vs. no)	1.341	0.92-1.938	0.118	1.660	1.105-2.495	0.015
MELD (per point increase)	1.065	1.039-1.091	<0.001	1.067	1.039-1.095	<0.001
MAC (per cm increase)	0.998	0.995-1.001	0.181	1.004	0.999-1.008	0.109
MAMC (per cm increase)	0.996	0.992-0.999	0.020	0.996	0.992-1.000	0.026
TSFT (per mm increase)	0.985	0.967-1.003	0.097	0.992	0.957-1.027	0.642
SGA						
A (Ref)	1			1		
B	1.403	0.962-2.045	0.079	1.333	0.836-2.126	0.227
C	1.663	1.010-2.738	0.045	1.419	0.723-2.785	0.309
Metabolism						
Normometabolic (Ref)	1			1		
Hypometabolic	1.258	0.553-2.860	0.584	1.566	0.678-3.621	0.294
Hypermetabolic	2.070	1.211-3.537	0.008	2.618	1.457-4.705	0.001
